# Clinical characteristics and outcomes of pediatric cancer patients admitted to the intensive care unit during treatment: a retrospective analysis

**DOI:** 10.3389/or.2026.1699224

**Published:** 2026-05-01

**Authors:** Joanna Sordyl, Maria Damps, Katarzyna Skowron-Kandzia, Karolina Baranowska, Agnieszka Mizia-Malarz

**Affiliations:** 1 Department of Pediatrics, Pediatric Oncology, Hematology and Chemotherapy Unit, Upper Silesian Child Health Centre, Medical University of Silesia, Katowice, Poland; 2 Department of Anesthesiology and Intensive Care, Upper Silesian Child Health Centre, Medical University of Silesia, Katowice, Poland; 3 Prenatal Diagnostic and Genetic Clinic, Medical University of Silesia, Zabrze, Poland

**Keywords:** cancers, pediatric intensive care, pediatric oncology, septic shock, treatment

## Abstract

**Background:**

Treatment of childhood cancer is associated with a substantial risk of life-threatening complications that may require admission to a pediatric intensive care unit (PICU). Data describing clinical characteristics, indications for intensive care, and outcomes in this population remain limited.

**Methods:**

This retrospective, single-center study analyzed all pediatric oncology patients who required PICU admission because of acute clinical deterioration during cancer treatment between 2010 and 2022. Fifty-one children treated at the Department of Pediatric Oncology, Hematology, and Chemotherapy of the Upper Silesian Child Health Centre (Medical University of Silesia, Katowice, Poland) were included. Demographic data, underlying malignancies, reasons for PICU admission, laboratory findings, therapeutic interventions, and outcomes were analyzed.

**Results:**

A total of 61 PICU hospitalizations occurred, including repeated admissions in nine patients with leukemia. Most children were diagnosed with hematological malignancies, followed by brain tumors and extracranial solid tumors. The leading indications for PICU admission were septic shock, respiratory failure, and cardiopulmonary failure, accounting for 75.4% of cases. Cardiovascular support with catecholamines was required in 52.5% of patients, and renal replacement therapy was initiated in 18.0%. Both catecholamine use and renal replacement therapy were independently associated with a significantly increased risk of mortality. Life-threatening complications requiring intensive care occurred most frequently during leukemia treatment.

**Conclusion:**

Severe treatment-related complications requiring intensive care are common in pediatric oncology, particularly among children with hematological malignancies. Septic shock during neutropenia remains the predominant cause of PICU admission. Early identification of clinical deterioration and clearly defined criteria for timely PICU referral—especially in cases of septic shock—may improve survival outcomes in this vulnerable population.

## Introduction

Childhood cancer represents a major global medical and public health challenge. Approximately 400,000 individuals younger than 20 years are diagnosed with cancer each year worldwide, making malignancy the leading cause of non-communicable disease-related mortality in children and adolescents (1). Pediatric cancers constitute a heterogeneous group of diseases that include hematological malignancies, intracranial and extracranial solid tumors, which differ substantially in incidence, age at diagnosis, treatment strategies, and prognosis. Over the past 5 decades, significant advances in diagnostics, multimodal therapy, and supportive care have markedly improved survival outcomes for children with cancer in high-income countries, where overall survival now approaches 80% (2). These improvements have enabled the treatment of more advanced disease stages but have also increased exposure to intensive therapies associated with serious, potentially life-threatening complications. As a result, children undergoing cancer treatment are at high risk of infectious, hematologic, metabolic, neurologic, and cardiopulmonary complications.

Approximately 40% of pediatric oncology patients require admission to an intensive care unit at least once during the course of their treatment (3). Pediatric intensive care units (PICUs) therefore represent an integral component of comprehensive oncologic care. Timely access to intensive care and appropriate management of acute complications may substantially influence both short-term survival and long-term outcomes in this population.

Despite the critical role of intensive care in pediatric oncology, data describing the clinical profile, reasons for PICU admission, and outcomes of children with cancer remain limited, particularly in European settings (4). Understanding the factors associated with clinical deterioration and mortality in pediatric oncology patients requiring intensive care is essential to optimize referral strategies, improve early recognition of critical illness, and refine treatment pathways.

The aim of this study was to characterize the clinical features, indications for PICU admission, therapeutic interventions, and outcomes of children with cancer requiring intensive care during treatment at a tertiary pediatric oncology center.

## Methods

### Study design and population

This retrospective, observational, single-center study was conducted at the Department of Pediatric Oncology, Hematology, and Chemotherapy and the Pediatric Anesthesiology and Intensive Care Unit of the Upper Silesian Child Health Centre, Medical University of Silesia, Katowice, Poland. The study period spanned from January 2010 to December 2022.

Among 896 children diagnosed with cancer and treated at the institution during the study period, 51 patients (5.7%) required admission to the pediatric intensive care unit (PICU) due to acute, life-threatening clinical deterioration and were included in the analysis. All PICU admissions occurring during active oncologic treatment were eligible for inclusion. No exclusion criteria were applied. Repeated PICU admissions of the same patient were analyzed as independent events, reflecting distinct episodes of clinical deterioration.

Patients were categorized according to the underlying malignancy into three diagnostic groups: hematological malignancies (HM), central nervous system tumors (BT), and extracranial solid tumors (ST). For comparative analyses, BT and ST groups were also combined into a single solid tumor group (T), given overlapping clinical characteristics and patterns of complications.

### Ethical approval

The study protocol was approved by the Ethics Committee of the Medical University of Silesia (PCN/CBN/0052/44/23). Given the retrospective nature of the study and the use of anonymized archival data, the requirement for informed consent was formally waived. The study was not classified as a medical experiment and was conducted in accordance with applicable institutional and national ethical guidelines.

### Data collection

Clinical data were extracted retrospectively from electronic and paper medical records. Collected variables included demographic characteristics, underlying oncologic diagnosis, laboratory parameters, bacteriological culture results, causes of clinical deterioration, indications for PICU admission, therapeutic interventions during PICU stay, and patient outcomes.

Laboratory parameters analyzed were the last available results obtained in the oncology department prior to transfer to the PICU and included inflammatory markers, complete blood count indices, and relevant biochemical values. Bacteriological data were derived from blood, urine, and stool cultures obtained during the acute deterioration period.

Indications for PICU admission were categorized into respiratory failure, circulatory failure, neurologic deterioration (including seizures and altered consciousness), and other acute complications. Therapeutic interventions analyzed included invasive mechanical ventilation, catecholamine administration, renal replacement therapy, and length of PICU stay.

### Statistical analysis

Continuous variables were summarized using means with standard deviations or medians with ranges, as appropriate. Categorical variables were presented as absolute numbers and percentages. The Shapiro–Wilk test was used to assess normality of data distribution. As most continuous variables did not follow a normal distribution, comparisons between groups were performed using the Mann–Whitney U test. Categorical variables were compared using the χ^2^ test with Yates’ correction. Odds ratios (ORs) with 95% confidence intervals (CIs) were calculated to assess associations between selected clinical interventions—particularly catecholamine use and renal replacement therapy—and mortality. Statistical significance was defined as a p-value ≤0.05. Analyses were performed using Statistica 13 (StatSoft, Tulsa, OK, USA) and PQStat software (version 1.8.4.162).

## Results

### Patient characteristics

Between 2010 and 2022, 51 children with active malignancy required admission to the pediatric intensive care unit (PICU). The cohort included 19 girls (37.3%) and 32 boys (62.7%), with a mean age of 7.68 ± 5.62 years (median 5.75 years). During the study period, a total of 61 PICU hospitalizations occurred; eight patients with leukemia were admitted twice, and one patient was admitted three times. Each hospitalization was analyzed as an independent event.

Among all PICU hospitalizations, hematological malignancies accounted for 40 cases (65.6%), while solid tumors accounted for 21 cases (34.4%), including 10 central nervous system tumors (16.4%) and 11 extracranial solid tumors (18.0%). There were no significant differences between diagnostic groups with respect to sex distribution or age when analyzed separately. However, when solid tumors were analyzed as a combined group, children with solid tumors were significantly younger than those with hematological malignancies (p = 0.025) (Table 1).

**TABLE 1:** Characteristic of the study population.

Parameter	Group HM	Group T	Total	p
Gender				
Girls [n]	16	8	24	0.884
Boys [n]	24	13	37	0.884
Age				
[Years]±SD, median	8.923 ± 5.608, 9.035	5.317 ± 4.951, 4.83	7.681 ± 5.621, 5.75	0.025

Group HM, hematological malignances, group T–solid tumors.

### Indications for PICU admission

The leading indications for PICU admission across the entire cohort were respiratory failure, circulatory failure, and neurologic deterioration. Respiratory failure was the most common reason for admission in both the hematological malignancy (HM) group (n = 17, 42.5%) and the solid tumor (T) group (n = 7, 33.3%).

**FIGURE 1:**
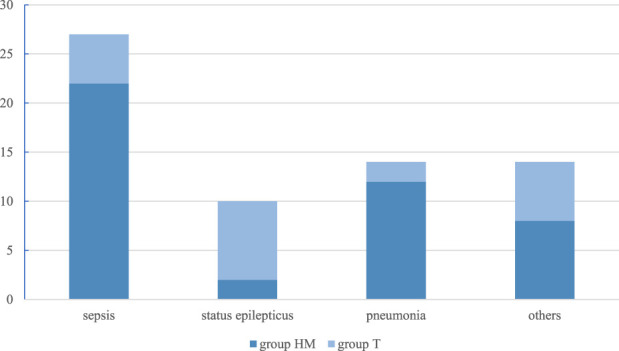
Main causes [n] of health deterioration in the subgroups. group HM - hematological malignancies; group T - solid tumors.

**FIGURE 2:**
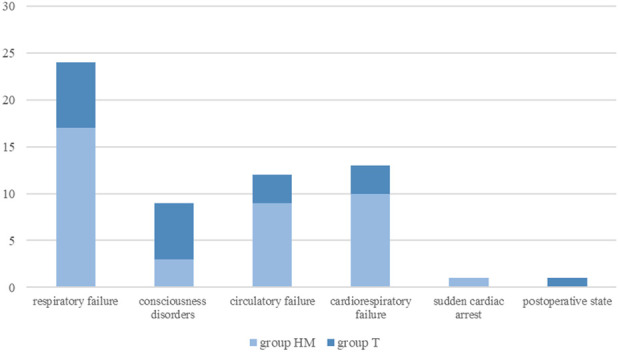
Most common causes of PICU admission in the subgroups. group HM - hematological malignancies; group T - solid tumors; PICU, pediatric intensive care unit.

Circulatory failure occurred predominantly in children with hematological malignancies (n = 10, 25%), whereas neurologic deterioration—primarily impaired consciousness and seizures—was more frequently observed in children with solid tumors (n = 6, 28.6%) (Figures 1, 2). The distribution of indications for PICU admission differed significantly between the HM and T groups (p < 0.05).

### Infectious complications and laboratory findings

Infections were the dominant cause of clinical deterioration, affecting 46 patients (75.4%). Infectious complications were significantly more frequent in children with hematological malignancies compared with those with solid tumors (85.0% vs. 57.1%). In contrast, status epilepticus was the predominant cause of deterioration in the solid tumor group (38.1%), particularly among children with central nervous system tumors. Laboratory parameters differed significantly between diagnostic groups. Children with hematological malignancies demonstrated significantly higher inflammatory markers and more profound cytopenias, including lower leukocyte, neutrophil, hemoglobin, and platelet counts, compared with children with solid tumors (Table 2). The severity of cytopenia was positively correlated with concentrations of C-reactive protein and procalcitonin.

**TABLE 2:** Laboratory parameters of the study population.

Parameter	Group HM	Group T	Total	p
CRP [mg/L]±SD, median	142.415 ± 104.417, 124.9	69.2 ± 91.567, 27.95	117.192 ± 105.82, 93	0.002
PCT [ng/mL] ±SD, median	33.085 ± 34.934, 26.5	7.463 ± 23.443, 0.0	23.936 ± 33.483, 2.8	<0.05
WBC [*10^3^/ul] ±SD, median	13.191 ± 73.183, 0.6	7.105 ± 6.704, 5.845	11.154 ± 59.201, 1.1	<0.05
ANC [*10^3^/ul] ±SD, median	1.630 ± 4.782, 0.0	4.859 ± 5.478, 3.0	2.760 ± 5.226, 0.115	<0.05
HGB [g/dL] ±SD, median	8.897 ± 1.377, 8.95	10.654 ± 1.899, 10.3	9.549 ± 1.782, 942	<0.05
PLT [*10^3^/ul] ±SD, median	47.3 ± 106.344, 27.0	247.773 ± 241.911, 124	119.393 ± 193.087, 40	<0.05

CRP- C, reactive protein; PCT, procalcitonin; WBC, white blood cell; ANC, neutrophil count; HGB, hemoglobin; PLT, thrombocytes.

### Microbiological findings

Bacterial cultures were obtained in 61 hospitalizations. Positive cultures were identified in 35 cases (57.4%), while no significant bacterial growth was detected in 26 cases (42.6%). Positive blood cultures were significantly more frequent in children with hematological malignancies than in those with solid tumors (27 vs. 8; p = 0.02) (Table 3).

**TABLE 3:** Bacterial culture results in study population.

Bacterial culture	Study population	Total	p
Positive	n = 35	n = 61	—
Negative	n = 26		

Bacterial culture	Group HM	Group T	Total	p
Positive	n = 27	n = 8	n = 35	0.02
Negative	n = 13	n = 13	n = 26	

Gram-negative bacteria predominated, with extended-spectrum β-lactamase (ESBL)-producing *Klebsiella pneumoniae* (26.3%) and ESBL-producing *Escherichia coli* (15.8%) being the most frequently isolated pathogens. Fungal infections, confirmed by blood culture or polymerase chain reaction, occurred exclusively in children with hematological malignancies (n = 7), with mixed bacterial–fungal infections observed in a subset of patients.

### PICU interventions and length of stay

The mean length of PICU stay was 17.6 ± 20.2 days (median 10.5 days), with no statistically significant differences between diagnostic groups. The majority of patients required invasive mechanical ventilation (n = 54, 85.5%), most frequently initiated on the first day of PICU admission (87.1%). Rates of intubation and timing of ventilation did not differ significantly between groups.

Cardiovascular support with catecholamines was required in 32 hospitalizations (52.5%). Renal replacement therapy for acute kidney injury was initiated in 11 cases (18.0%), primarily among children with hematological malignancies. The average duration of renal replacement therapy was 1.76 ± 8.67 days, with no significant intergroup differences.

### Outcomes

Overall PICU mortality was 29.5% (18 deaths). Mortality was higher in the hematological malignancy group (32.5%) than in the solid tumor group (23.8%), although this difference did not reach statistical significance. The most common cause of death was cardiorespiratory failure.

The use of catecholamines during PICU treatment was strongly associated with increased mortality risk (OR = 15.3; 95% CI, 2.65–396.70; p = 0.006). Similarly, renal replacement therapy was associated with a significantly higher risk of death (OR = 5.34; 95% CI, 1.04–31.25; p = 0.006). Mortality was not significantly associated with age, underlying malignancy type, or the presence of positive bacterial cultures.

### Follow-up

Three-year survival was assessed for the entire cohort. Of the 51 patients, 30 (58.8%) died within 2 years of PICU hospitalization. Among these, 18 deaths (60%) occurred during the PICU stay, while 12 patients (40%) died due to progression of the underlying malignancy. Overall, 21 patients (41.2%) survived for at least 3 years following PICU admission.

## Discussion

This study characterizes critically ill pediatric oncology patients requiring PICU admission during active cancer treatment and identifies key clinical patterns associated with poor outcomes. Life- threatening complications occurred most frequently in children with hematological malignancies and were predominantly driven by infectious etiologies, particularly septic shock during neutropenia. The need for catecholamine support and renal replacement therapy emerged as the strongest predictors of mortality, underscoring the severity of organ dysfunction at the time of PICU admission.

### Clinical deterioration in pediatric oncology patients

Children with hematological malignancies represented the majority of PICU admissions in this cohort, consistent with previous reports (4–7). The systemic nature of these diseases, combined with intensive chemotherapy, predisposes patients to profound cytopenias, severe infections, and rapid clinical deterioration (8, 9). In the present study, children with hematological malignancies exhibited significantly higher inflammatory markers and more severe leukopenia, neutropenia, anemia, and thrombocytopenia compared with those with solid tumors. The positive correlation between cytopenia severity and inflammatory markers highlights the vulnerability of this population to overwhelming infection and sepsis (10, 11). Infectious complications were the leading cause of PICU admission, accounting for more than three-quarters of cases. Septic shock during neutropenia was particularly prominent and remains a central challenge in pediatric oncology. Gram-negative pathogens, especially ESBL-producing *Klebsiella pneumoniae* and *Escherichia coli*, predominated, reflecting the high-risk microbiological profile in immunocompromised children and emphasizing the importance of early antimicrobial therapy and vigilant monitoring (12).

### Organ support as a marker of severity and prognosis

The requirement for invasive mechanical ventilation was notably high, with most patients intubated within the first day of PICU admission. This finding suggests that respiratory failure often developed rapidly and was severe at presentation. Hypoxemia in this population may reflect a combination of infectious lung injury, systemic inflammatory response, capillary leak, and secondary effects of circulatory or renal failure (8, 13, 14). Compared with other published series, the proportion of intubated patients in this study was high, indicating that many children were admitted to the PICU with advanced respiratory compromise (8, 15, 16). Catecholamine use was required in more than half of PICU admissions and was strongly associated with mortality. Similarly, renal replacement therapy—used in nearly one-fifth of patients—was independently associated with an increased risk of death. Clinically, these interventions reflect the presence of refractory shock and multi-organ failure rather than isolated organ dysfunction. Their strong association with mortality reinforces their value as prognostic indicators in critically ill pediatric oncology patients.

Importantly, mortality was not significantly associated with age, type of malignancy, or microbiological confirmation of infection. This suggests that physiologic severity at the time of intensive care, rather than baseline oncologic diagnosis alone, is the principal determinant of short-term outcome (17–19).

### Neurologic deterioration and tumor-specific risks

Neurologic deterioration, including impaired consciousness and status epilepticus, was more common in children with solid tumors, particularly central nervous system tumors. These findings are consistent with tumor-related complications, treatment-related neurotoxicity, and intracranial hypertension. In contrast to infectious complications in hematological malignancies, neurologic emergencies in children with solid tumors may require a different clinical surveillance strategy focused on early recognition of neurologic signs and rapid escalation of care (8).

### Implications for clinical practice and early ICU referral

Taken together, these findings highlight the importance of early recognition of clinical deterioration in pediatric oncology patients. The high mortality associated with catecholamine use and renal replacement therapy suggests that outcomes may be improved if patients are transferred to the PICU before progression to advanced shock or multi-organ failure.

Clinical tools such as the Pediatric Early Warning System (PEWS) may support earlier identification of deterioration by integrating vital signs and clinical observations into structured decision-making (20). While PEWS does not replace clinical judgment, its use may facilitate timely escalation of care, particularly in neutropenic children with evolving sepsis or respiratory compromise (21). Earlier PICU admission may allow the initiation of non-invasive ventilation, aggressive fluid and antimicrobial management, and closer hemodynamic monitoring before irreversible organ damage occurs (22).

## Limitations

This study has limitations inherent to its retrospective, single-center design. The relatively small sample size limits the ability to perform multivariable analyses or identify subtler predictors of outcome. Additionally, decisions regarding PICU admission and initiation of organ support were made by treating clinicians and may have varied over time. Despite these limitations, the study provides clinically relevant insights into deterioration patterns and prognostic markers in pediatric oncology patients requiring intensive care.

## Data Availability

The datasets presented in this study can be found in online repositories. The names of the repository/repositories and accession number(s) can be found below: https://data.mendeley.com/datasets/t46w53nxct/1.
